# Association between Stress and White Matter Hyperintensity Volume in Diverse Populations: A HABS‐HD Study

**DOI:** 10.1002/alz.093446

**Published:** 2025-01-09

**Authors:** Antoine R Trammell, James J. Lah, Monica W Parker, Felicia Goldstein, Chadwick M Hales, Crystal P Davis, Cornelya D Dorbin, Sabria Saleh, Elizabeth Murphy‐Tchivzhel, Alecia Michella Rose, Amber Osborne, Adiva Ahmad, Jahmila Al‐Amin, Sid E. O'Bryant

**Affiliations:** ^1^ Emory University Goizueta Alzheimer's Disease Research Center, Atlanta, GA USA; ^2^ Emory University, Atlanta, GA USA; ^3^ Emory University School of Medicine, Atlanta, GA USA; ^4^ University of North Texas Health Science Center, Fort Worth, TX USA

## Abstract

**Background:**

Older African American (AA) and Hispanic American (HA) adults have a higher risk for Alzheimer’s disease and related dementias (ADRD) than older non‐Hispanic white (NHW) adults. Adverse experiences like stress and discrimination may contribute to the higher vascular disease burden observed in AA and HA persons. Cerebrovascular disease reflected by white matter hyperintensity (WMH) lesions on magnetic resonance imaging (MRI) may affect ADRD risk in older AA and HA adults. As stress may influence vascular disease and thus ADRD risk, this study explores associations between stress and WMH burden in a diverse cohort.

**Method:**

We analyzed stress scores and WMH volume data from self‐reported NHW, AA, and HA participants in the Health and Aging Brain Study: Health Disparities. Data obtained at the baseline study visit includes the Clinical Dementia Rating (CDR), Chronic Stress Scale (CSS), Everyday Discrimination Scale (EDS), Penn State Worry Questionnaire (PSWQ), Geriatric Depression Scale (GDS), and MRI brain scans. With this data, we used global CDR scores (normal=0, impaired≥0.5) and race to create race/cognitive groups (white/normal, white/impaired, AA/normal, AA/impaired, HA/normal, HA/impaired). We compared baseline characteristics, stress and GDS scores, and WMH burden in the groups with ANOVA and chi‐square. Next, we performed linear regression adjusted for age, education, and sex to test associations between stress scores and WMH burden.

**Result:**

Data from 3,035 participants was analyzed (mean age: 65.2; female: 62.3%; impaired: 20.1%). Compared to the NHW and HA groups, AA participants had higher CSS scores (NHW: 7.44, HA: 7.1, AA: 8.42, p=.0003), EDS scores (NHW: 5.59, HA: 6.2, AA: 9.47, p<.0001), and WMH volume (NHW: 4.16, HA: 3.18, AA: 18.4, p<.0001). The HA group had higher GDS scores (NHW: 4.86, HA: 6.71, AA: 5.61, p<.0001). Regression showed that higher EDS scores were associated with higher WMH volume in the AA/impaired (β=3.48, p=.02) and HA/impaired (β=.27, p=.002) groups.

**Conclusion:**

Our results suggest that higher discrimination scores may be linked to higher WMH volume in older AA and HA adults with cognitive impairment. As vascular disease may contribute to their higher ADRD risk, further study of stress, microvascular brain disease, and ADRD risk is needed.

